# Positive susceptibility‐based contrast imaging with dephased balanced steady‐state free precession

**DOI:** 10.1002/mrm.30421

**Published:** 2025-03-13

**Authors:** Jonas Frederik Faust, Peter Speier, Axel Joachim Krafft, Sunil Patil, Ravi Teja Seethamraju, Mark E. Ladd, Florian Maier

**Affiliations:** ^1^ Faculty of Physics and Astronomy Ruprecht‐Karls‐Universität Heidelberg Heidelberg Germany; ^2^ New Markets, Magnetic Resonance Siemens Healthineers AG Erlangen Germany; ^3^ Research & Clinical Translation Magnetic Resonance, Siemens Healthineers AG Erlangen Germany; ^4^ Siemens Medical Solutions USA Inc. Malvern Pennsylvania USA; ^5^ Medical Physics in Radiology German Cancer Research Center Heidelberg Germany; ^6^ Faculty of Medicine Ruprecht‐Karls‐Universität Heidelberg Heidelberg Germany

**Keywords:** bSSFP, dephased MRI, interventional MRI, positive contrast, white‐marker imaging

## Abstract

**Purpose:**

Dephasing gradients can be introduced within a variety of gradient‐echo pulse sequences to delineate local susceptibility changes (“White‐Marker” phenomenon), e.g., for the visualization of metallic interventional devices which are otherwise difficult to display. We investigated dephased balanced steady‐state free precession (d‐bSSFP) and compared it with similar contrast techniques: dephased RF‐spoiled fast low‐angle shot (d‐FLASH) and dephased steady‐state free precession (d‐SSFP).

**Methods:**

A signal model was formulated to describe the positive contrast in d‐bSSFP. For the example of an MR‐compatible aspiration needle, the positive contrast artifact appearance was theoretically derived, and the model was verified in a water phantom at B_0_ = 0.55 T. Model accuracy was evaluated by comparing the measured artifact size (for TEs between 3.4 ms and 50 ms) and the signal magnitude to the model prediction.

**Results:**

While positive contrast artifacts for d‐FLASH and d‐SSFP are axisymmetric with respect to the generating object, for d‐bSSFP, a point‐symmetric susceptibility artifact arises for a cylindrical needle due to the characteristic signal formation. The observed d‐bSSFP artifact size was in accordance with the model (error < 1 mm). Measured (predicted) cumulated artifact signal was 1.13 ± 0.07 (1.27) times higher and 5.9 ± 0.4 times higher than the d‐SSFP and d‐FLASH cumulated artifact signal, respectively. In contrast to d‐SSFP, the d‐bSSFP artifact was robust against banding artifacts.

**Conclusion:**

d‐bSSFP contrast is well described by the introduced model. Positive contrast artifacts show higher cumulated signal magnitude, symmetry, and homogeneity compared with d‐FLASH and d‐SSFP and can therefore improve device visualization and potentially device localization.

## 1 INTRODUCTION

In MR‐guided interventions, metallic interventional devices can locally disturb the magnetic field, creating a characteristic device artifact in the MR image which is often used for passive device localization.^1^, ^2^, ^3^, ^4^, ^5^ Due to the difference in magnetic susceptibilty of metallic interventional devices and the surrounding tissue, a local magnetic field gradient is induced. In conventional gradient‐echo (GRE)–based imaging, this leads to dephasing of local magnetization, and therefore signal loss.^6^, ^7^, ^8^ As signal voids in MR images are ambiguous and large signal voids near devices can impede localization, several methods for the generation of susceptibilty‐based, positive contrast have been proposed in the past.^9^, ^10^, ^11^, ^12^, ^13^, ^14^, ^15^ These methods make use of the distortion in the magnetic field induced by the metallic device to generate high signal intensities in proximity of the interventional device.

A prominent example of these positive susceptibility‐based contrast techniques is so‐called White‐Marker (WM) imaging.^9^ By applying an additional external gradient moment before image readout, the local susceptibilty‐induced gradient moment in proximity of the device can be counteracted, reestablishing phase coherence of the local magnetization, and therefore restoring signal. Simultaneously, the external gradient dephases the signal further away from the device where the magnetic field is not disturbed by the device susceptibilty, hence suppressing anatomy in the image and exclusively highlighting the device. WM imaging has been used for the localization of paramagnetic markers and interventional devices.^9^, ^16^ A comparable gradient‐compensating approach has also been used to create positive contrast of superparamagnetic iron oxide particle (SPIO)–labeled cells.^17^ In the original work by Seppenwoolde et al., WM contrast was established by shortening the slice‐selection rephaser gradient and incidentally creating a residual excess WM gradient moment in the slice‐selection direction to counteract the local susceptibilty‐induced gradient moment.^9^ Bakker et al. later generalized the concept of gradient dephasing to arbitrary gradient directions (corresponding to discrete shifts of the acquisition k‐space) and established the term “dephased MRI”.^10^

In general, the generation of dephased contrast is primarily possible with GRE‐type sequences, as susceptibility‐induced dephasing is rephased with spin‐echo sequences. GRE‐type sequences typically comprise radiofrequency (RF)‐spoiled GRE or also called spoiled fast low‐angle shot (FLASH), steady‐state free precession (SSFP), and balanced SSFP (bSSFP).^18^ Whereas the original paper by Seppenwoolde et al. used dephased spoiled FLASH contrast,^9^ which we termed d‐FLASH in the context of this paper, Patil et al. introduced “echo‐dephased SSFP”,^15^ which we will call dephased SSFP (d‐SSFP) for simplicity. Dephased bSSFP (d‐bSSFP) has also been used in the past by some authors for delineation of interventional catheters and needles.^19^, ^20^ Some preliminary experimental work has been carried out to characterize the d‐bSSFP contrast and found it favorable in terms of achievable signal intensity compared with d‐FLASH.^21^ However, d‐bSSFP has never been formally introduced or characterized, nor has the signal mechanism been compared with d‐FLASH or d‐SSFP.

In this work, we provide a comprehensive investigation of d‐bSSFP contrast. We introduce a signal model for d‐bSSFP and compare the mechanism of positive signal realization with the related contrast mechanisms of d‐FLASH and d‐SSFP. Using an aspiration biopsy needle as an example, we derive an analytical description of the device artifact that we validate in phantom measurements.

## 2 THEORY

**FIGURE 1:**
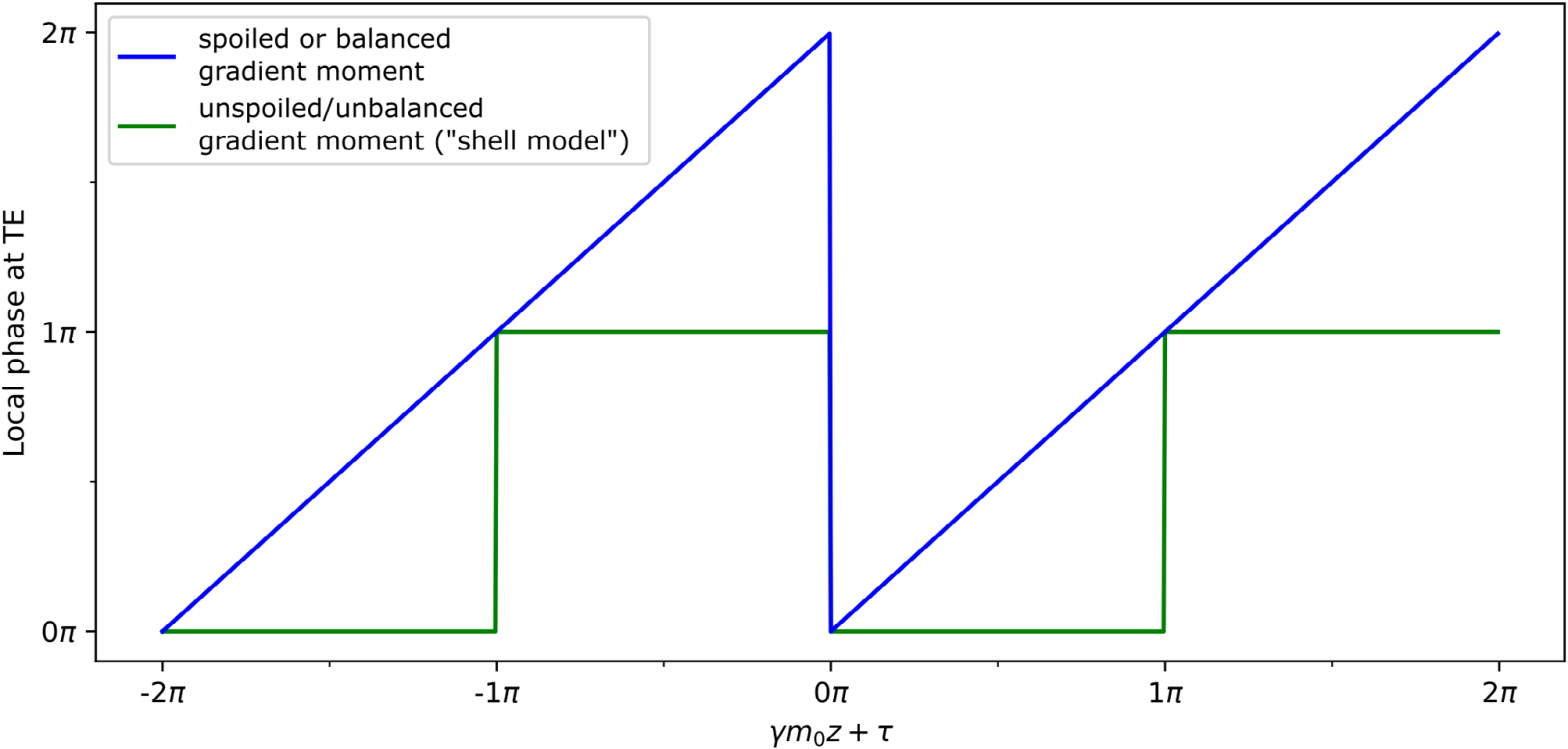
Phase of local magnetization at TE for an unspoiled/unbalanced or a spoiled or balanced gradient moment $m_{0}$ acting along z‐direction with a constant phase offset τ. For spoiled or balanced gradients, the phase of the local magnetization at TE depends linearly on $m_{0}$. For an unspoiled/unbalanced gradient, the local magnetization rephases at TE, either at 0 or π (“shell model”^23^, requiring an absolute induced gradient moment of $2m_{0}$ at the end of TR).

Let *G*(*t*) be a magnetic field gradient that generates a linear field variation in z‐direction (without loss of generality), acting on local magnetization at time *t* during the repetition time (TR). At the echo time (TE), the gradient will have introduced a gradient moment $m_{0}=\int_{0}^{\mathrm{TE}}G\left(t\right)\;dt$. At the end of TR, effects of an acting gradient moment on the local magnetization can either be spoiled (disruption of transverse coherences) or balanced (zero net gradient moment at the end of TR), or remain unspoiled/unbalanced. For spoiled or balanced gradient moments, local phase at TE will depend linearly on $m_{0}$. For unspoiled/unbalanced gradient moments, phase at TE relates to the absolute induced gradient moment across TR in a complex fashion that is dependent on T_1_, T_2_, and the flip angle.^22^ For gradients acting uniformly across TR (in the sense that the absolute gradient moment at the end of TR equals $2m_{0}$), Lebel et al. proposed to model the signal phase at TE with a simple square wave (SW) function (“shell model”).^23^ The phase introduced at TE to the local magnetization at position z for unspoiled/unbalanced as well as for spoiled or balanced gradients is therefore given by

(1) $$\begin{array}{ll} & \phi_{\text{spoiled or balanced}}\left(z,m_{0},\tau \right)=\gamma m_{0}z+\tau \\ & \phi_{\text{unspoiled}/\text{unbalanced}}\left(z,m_{0},\tau \right)=\mathrm{SW}\left(\gamma m_{0}z+\tau \right)\\ & =\left\{\begin{array}{l} \pi \;\text{for}\left(2k-1\right)\pi <\gamma m_{0}z+\tau <2k\pi \\ 0\;\text{for}\;2k\pi <\gamma m_{0}z+\tau <\left(2k+1\right)\pi \end{array}\right., \end{array}$$

where γ is the gyromagnetic ratio, k∈ℤ, and τ is a discrete phase offset. Figure 1 shows how unspoiled/unbalanced and spoiled or balanced gradients, acting uniformly during TR, influence the phase at TE according to Eq. (1).

The signal magnitude $S_{\text{voxel}}$ of a voxel with edge length Δz and unit length in x‐ and y‐direction (neglecting relaxation effects) for an intravoxel phase distribution (in z‐direction) is generically described as follows^10^:

(2) $$S_{\text{voxel}}=S_{\mathrm{SS}}\left|\int \text{rect}\left(\frac{z}{\Delta z}\right)e^{‐\mathrm{i\phi}\left(z,m_{0},\tau \right)}dz\right|,$$

where rect is the rectangular function (rect(x) = 1 for |x|<0.5; rect(x) = 0.5 for x = ±0.5; rect(x) = 0 elsewhere), *ϕ* is the phase of the local magnetization and $S_{\mathrm{SS}}$ describes the (location‐independent) steady‐state signal magnitude per voxel. The value of $S_{\mathrm{SS}}$ depends on the type of steady state that is established for the sequence (coherent steady‐state $S_{\mathrm{SSC}}$ for sequences without spoiling or incoherent steady‐state $S_{\mathrm{SSI}}$ for sequences with spoiling^24^). While both $S_{\mathrm{SSC}}$ and $S_{\mathrm{SSI}}$ depend on the tissue (spin density, T_1_, T_2_) and the chosen sequence parameters (TR, flip angle), $S_{\mathrm{SSC}}$ also varies with the locally induced phase advance during TR.^22^ In the context of the model and following Lebel et al.,^23^ we assume a constant coherent steady‐state signal magnitude $S_{\mathrm{SSC}}$, independent of the local phase advance during TR.

Seppenwoolde et al. investigated the emergence of bright contrast near metallic devices after the application of an external gradient $G_{\mathrm{WM}}$.^9^ They explained this effect, which they termed the WM phenomenon, as the consequence of local gradient moment compensation. A varying, susceptibility‐induced, magnetic field distortion near a metallic device results in a local susceptibility‐induced magnetic field gradient $G_{\text{susc}}$. For small voxels, we can linearize the induced field gradient (first‐order Taylor approximation of the magnetic field distortion). Considering an external WM gradient moment introducing a phase $\gamma m_{0,\mathrm{WM}}=2\pi /\Delta z$ at TE, we now investigate an underlying susceptibility‐induced gradient moment $m_{0,\text{susc}}$ (also assumed to act in z‐direction) and the additional external WM gradient acting simultaneously. We distinguish three cases: (i) $m_{0,\text{susc}}$ and $m_{0,\mathrm{WM}}$ both being spoiled at the end of TR (d‐FLASH); (ii) $m_{0,\text{susc}}$ and $m_{0,\mathrm{WM}}$ both being unspoiled/unbalanced at the end of TR (d‐SSFP); (iii) $m_{0,\text{susc}}$ being unspoiled/unbalanced and $m_{0,\mathrm{WM}}$ being balanced at the end of TR (d‐bSSFP). The type of the acting gradients for the different contrast methods is summarized in Table 1. Introducing the phase relations from Eq. (1) into Eq. (2), depending on the type of the acting gradients, we can calculate the average voxel signal magnitudes ${\tilde{S}}_{d‐\text{FLASH}}$, ${\tilde{S}}_{d‐\text{SSFP}}$, and ${\tilde{S}}_{d‐\text{bSSFP}}$:

(3) $$\begin{array}{ll} {\tilde{S}}_{d‐FLASH} & =S_{SSI}\left|\int \text{rect}\left(\frac{z}{\Delta z}\right)e^{-i(\gamma m_{0,susc}z+\frac{2\pi }{\Delta z}z)}dz\right|\\ {\tilde{S}}_{d‐SSFP} & =S_{SSC}\frac{\int_{0}^{2\pi }\left|\int \text{rect}\left(\frac{z}{\Delta z}\right)e^{-i\;\text{SW}(\gamma m_{0,susc}z+\frac{2\pi }{\Delta z}z+\tau )}dz\right|d\tau }{2\pi }\\ {\tilde{S}}_{d‐bSSFP} & =S_{SSC}\frac{\int_{0}^{2\pi }\left|\int \text{rect}(\frac{z}{\Delta z})e^{-i(\text{SW}(\gamma m_{0,susc}z+\tau )+\frac{2\pi }{\Delta z}z)}dz\right|d\tau }{2\pi } \end{array}$$

**TABLE 1:** Type of acting gradient moments in dephased MRI sequences (d‐FLASH, d‐SSFP, and d‐bSSFP). Two gradient moments (susceptibility‐induced and WM gradient moment) act simultaneously on the local magnetization. While spoiled or balanced gradient moments will introduce a linear phase at TE, local magnetization will rephase with a phase of 0 or π for unspoiled/unbalanced gradient moments (“shell model”^23^, see Eq. [1]).

	Susceptibilty‐induced gradient moment $m_{0,\text{susc}}$	White‐Marker gradient moment $m_{0,\mathrm{WM}}$
d‐FLASH	Spoiled	Spoiled
d‐SSFP	Unspoiled/unbalanced	Unspoiled/unbalanced
d‐bSSFP	Unspoiled/unbalanced	Balanced

**FIGURE 2:**
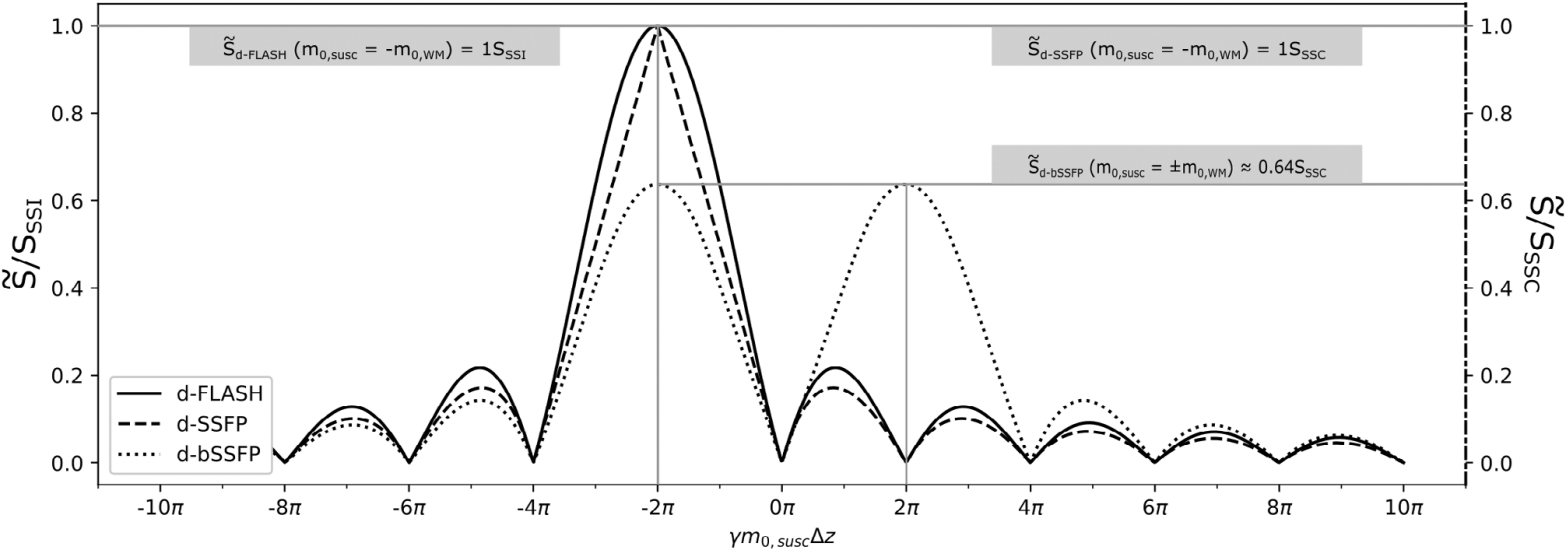
Average voxel signal magnitude for d‐FLASH, d‐SSFP, and d‐bSSFP contrast ($\gamma m_{0,\mathrm{WM}}=2\pi /\Delta z$) for a varying susceptibility‐induced gradient moment $m_{0,\text{susc}}$. The signal magnitude is calculated in relation to the normalized maximum signal magnitude of the underlying steady state, i.e., $S_{\mathrm{SSI}}$ for d‐FLASH and $S_{\mathrm{SSC}}$ for d‐SSFP and d‐bSSFP.

**FIGURE 3:**
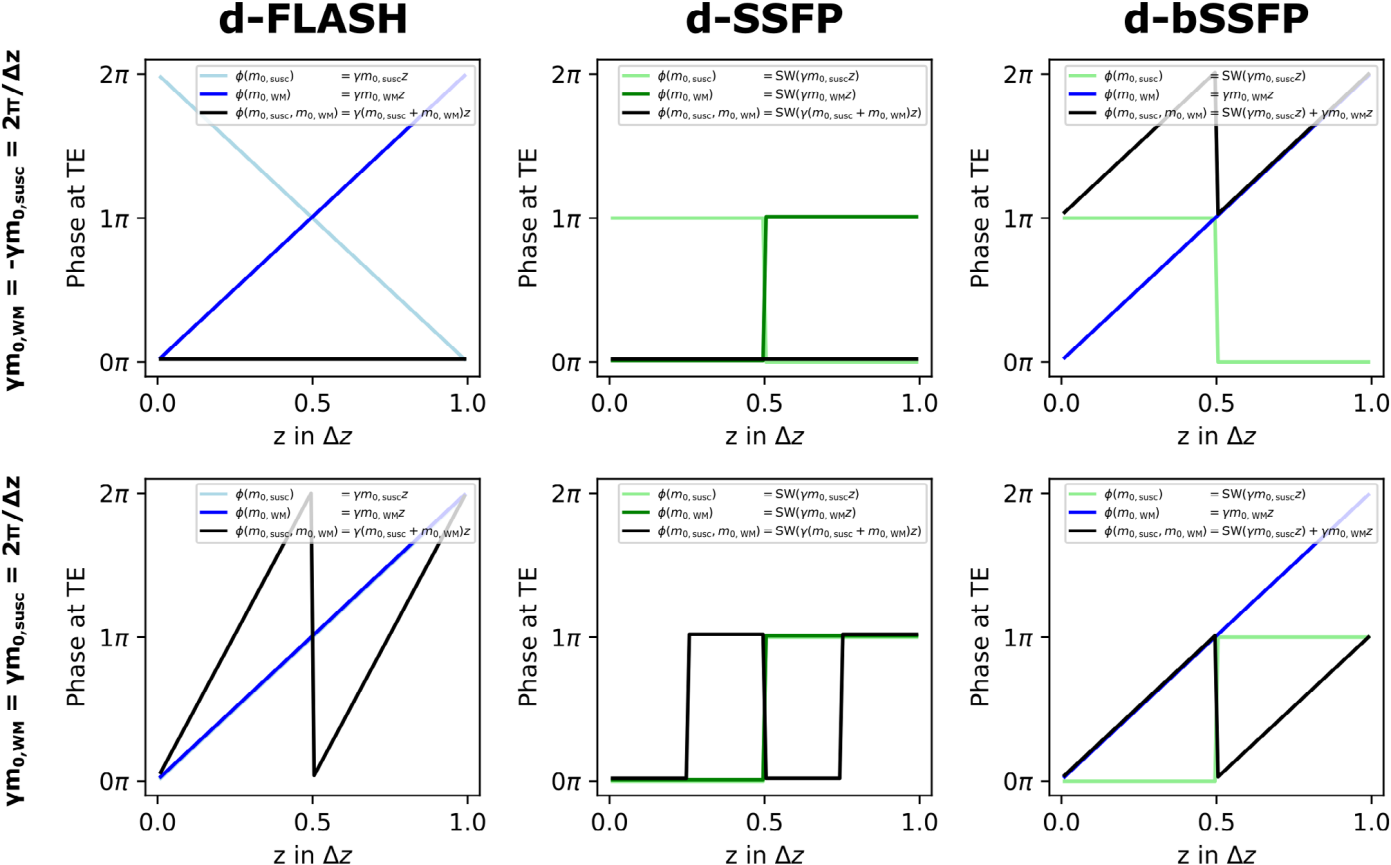
Intravoxel phase distribution ϕ of local magnetization at TE for d‐FLASH, d‐SSFP, and d‐bSSFP across one pixel (pixel edge length Δz) for the two cases: $\gamma m_{0,\mathrm{WM}}=-\gamma m_{0,\text{susc}}=2\pi ⁄\Delta z$ (expected signal maximum for d‐FLASH, d‐SSFP, and d‐bSSFP) and $\gamma m_{0,\mathrm{WM}}=\gamma m_{0,\text{susc}}=2\pi ⁄\Delta z$ (expected additional signal maximum for d‐bSSFP). The phase is plotted for $m_{0,\text{susc}}$ and $m_{0,\mathrm{WM}}$ acting independently (contributing phases following Eq. [1] with phase offset τ=0 ) as well as for both gradients acting simultaneously (summed resulting phase). Phase contributions from spoiled or balanced gradient moments are drawn in (light‐) blue and from unspoiled/unbalanced gradient moments in (light‐) green (overlapping lines are drawn with a slight offset for better visibility). For d‐FLASH and d‐SSFP, adding WM–induced and susceptibility‐induced phases leads to complete restoration of phase coherence for $m_{0,\text{susc}}=-m_{0,\mathrm{WM}}$. For d‐bSSFP, partial rephasing of the magnetization is achieved for $m_{0,\text{susc}}=\pm m_{0,\mathrm{WM}}$.

While for d‐FLASH, a discrete phase offset is not considered when integrating the signal across the voxel (as it only adds a constant factor that does not affect integrated signal magnitude), we have to average across a discrete phase offset τ for d‐SSFP and d‐bSSFP to calculate the average signal magnitude. A closed solution for the signal equations (up to first signal maximum at $\gamma m_{0,\text{susc}}=\pm 2\pi /\Delta z$ for ${\tilde{S}}_{d‐\text{bSSFP}}$ ) is given in the Appendix by Eq. (A1). Figure 2 shows the signal magnitude curves for the three investigated contrasts (${\tilde{S}}_{d‐\text{bSSFP}}$ numerically continued for $\left|\gamma m_{0,\text{susc}}\right|>2\pi /\Delta z$ ). The result for ${\tilde{S}}_{d‐\text{FLASH}}$ is the well‐known sinc function.^10^ The curve is shifted such that maximum signal appears at full gradient compensation ($\gamma m_{0,\mathrm{WM}}=-\gamma m_{0,\text{susc}}=2\pi /\Delta z$). For ${\tilde{S}}_{d‐\text{SSFP}}$, the signal maximum equally appears at full gradient compensation. Unlike for d‐FLASH and d‐SSFP, two instead of one signal maxima emerge for d‐bSSFP. Signal is maximal for $\gamma m_{0,\mathrm{WM}}=-\gamma m_{0,\text{susc}}=2\pi /\Delta z$, but also for $\gamma m_{0,\mathrm{WM}}=+\gamma m_{0,\text{susc}}=2\pi /\Delta z$ . While full $S_{\mathrm{SSI}}$ and $S_{\mathrm{SSC}}$ signal magnitude can be reached for d‐FLASH and d‐SSFP at maximum gradient compensation, only about 64% of the full $S_{\mathrm{SSC}}$ signal can be achieved with d‐bSSFP according to the model (see Eq. [A1]). For better illustration of the signal formation at the signal maxima, Figure 3 shows the intravoxel phase for $\gamma m_{0,\mathrm{WM}}=-\gamma m_{0,\text{susc}}$ , as well as $\gamma m_{0,\mathrm{WM}}=+\gamma m_{0,\text{susc}}$ for d‐FLASH, d‐SSFP, and d‐bSSFP. While for d‐FLASH and d‐SSFP, phase coherence is only reestablished for $m_{0,\text{susc}}$ and $m_{0,\mathrm{WM}}$ having opposite polarity, limited phase coherence is reestablished for d‐bSSFP independent of the respective gradient polarities.

## 3 METHODS

To evaluate d‐bSSFP contrast and compare it with d‐FLASH and d‐SSFP, we implemented three different research MR pulse sequences. Using a cylindrical aspiration needle as an example, we derived a description of the artifact (points of maximum signal intensity) with the introduced d‐bSSFP signal model. We verified it using an MR‐compatible needle placed in a water phantom.

### 3.1 MR pulse sequences

**FIGURE 4:**
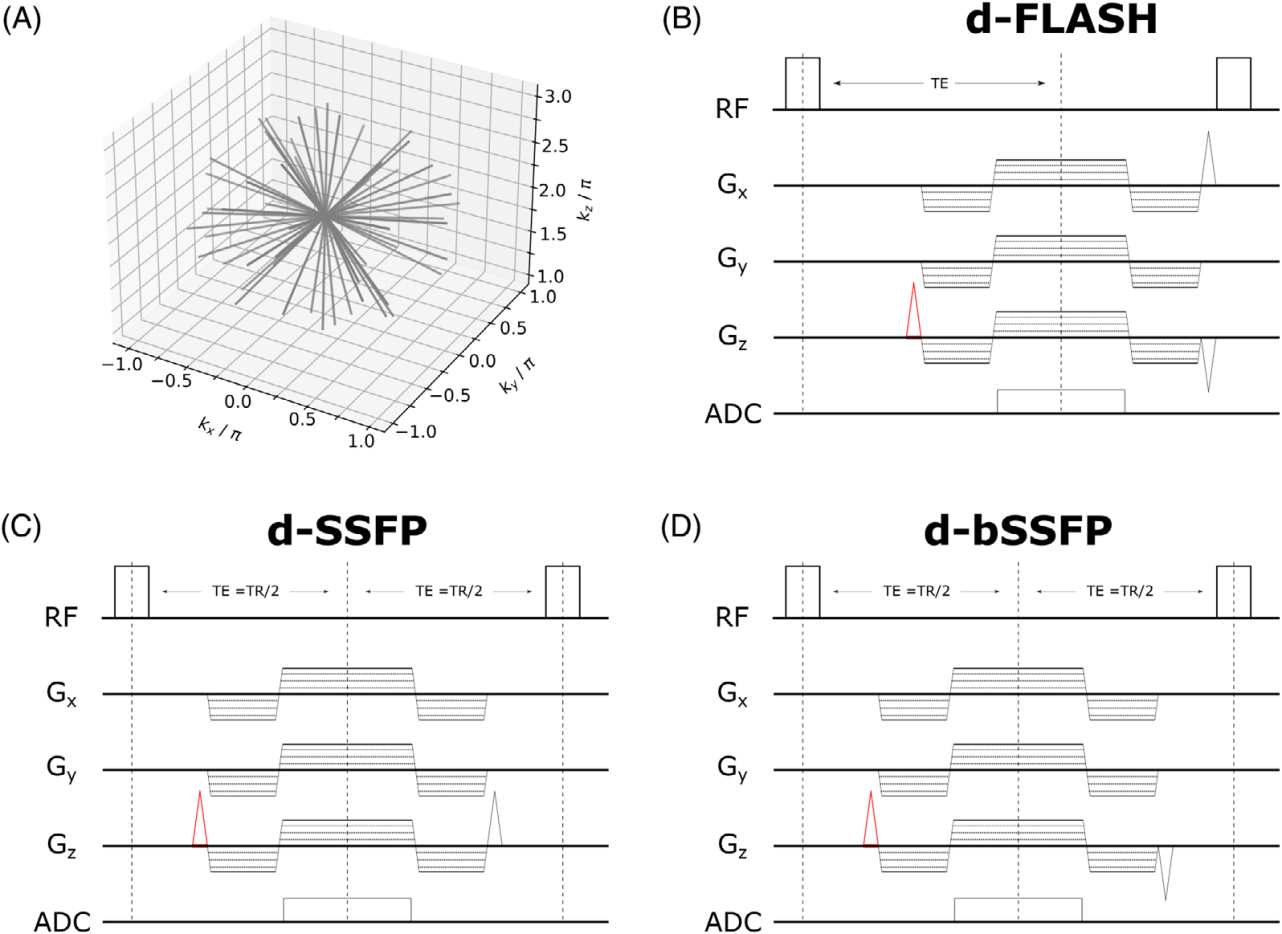
Example implementation of three MR pulse sequences to acquire images with d‐FLASH (B), d‐SSFP (C), and d‐bSSFP (D) contrast following a three‐dimensional radial k‐space trajectory (A). In all three sequence types, a WM gradient (red) shifts the acquired k‐space in z‐direction with $\gamma m_{0,\mathrm{WM}}=2\pi /\Delta z$ . For d‐FLASH contrast, all gradient moments are reverted, and a 2π‐spoiler gradient is played out in x‐direction at the end of TR (+ additional RF spoiling). For d‐SSFP contrast, an equal gradient moment to the WM gradient moment is played out at the end of TR. For d‐bSSFP, all gradient moments are balanced at the end of TR.

To generate d‐bSSFP, d‐FLASH, and d‐SSFP contrast, we implemented three research MR pulse sequences for 3D imaging (Figure 4B–D). A radial k‐space trajectory (Figure 4A) was chosen to mitigate potential distortion in the image caused by spatial frequency mismatch due to changed Larmor frequency near metallic devices (due to the changing frequency encoding direction of the acquired k‐space spokes, spatial frequency mismatch will lead to blurring rather than to cause discrete spatial shifts).^25^, ^26^ All three sequence types include a WM gradient, shifting the acquired k‐space in z‐direction with $\gamma m_{0,\mathrm{WM}}=2\pi /\Delta z$. To generate d‐FLASH contrast, the readout gradient is reverted at the end of each TR and a spoiler gradient inducing a dephasing of 2π per voxel is added in x‐direction with implemented RF spoiling.^27^, ^28^ To generate d‐SSFP contrast, an additional gradient moment equal to the WM gradient moment is played out after TE, leading to a net external gradient moment of $2m_{0,\mathrm{WM}}$ at the end of TR.^15^ Additionally, TE is fixed at TE = TR/2 to ensure that a net susceptibility‐induced gradient moment of $2m_{0,\text{susc}}$ acts across TR and Eq. (1) applies. To generate d‐bSSFP contrast, all played‐out gradient moments are balanced at the end of TR, including the WM gradient, and TE is again fixed at TE = TR/2. Image reconstruction using a NUFFT was implemented in Python with SigPy.^29^, ^30^

### 3.2 Contrast characterization for aspiration needle

**FIGURE 5:**
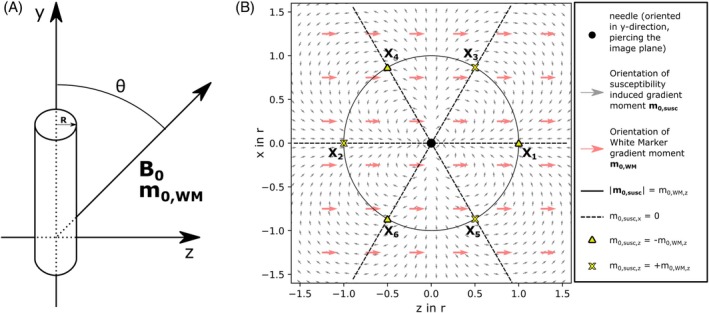
(A) Schematic of the infinite cylinder model, representing a needle, oriented in y‐direction in a right‐handed coordinate system. The $B_{0}$ field encloses an angle θ with the needle. The WM gradient moment $m_{0,\mathrm{WM}}$ is oriented parallel to the $B_{0}$ field. (B) The needle induces a local magnetic field gradient in the respective zx‐plane. The figure shows the needle piercing the image plane (black dot), and the orientation of the susceptibility‐induced field gradient and the WM field gradient (gray and red arrows, respectively). At radius r from the needle center as defined in Eq. (8) (solid black line), the induced gradient moment magnitude from the susceptibility‐induced field distortion matches the z‐component of the WM gradient moment magnitude. Along the dashed black lines, the x‐component of the susceptibility‐induced gradient moment $m_{0,\text{susc},x}$ vanishes. Using Eq. (9), we find three points (yellow triangles) with $m_{0,\text{susc},z}=-m_{0,\mathrm{WM},z}$ (points of maximum signal for d‐FLASH, d‐SSFP, and d‐bSSFP) and three points (yellow crosses) with $m_{0,\text{susc},z}=m_{0,\mathrm{WM},z}$ (additional points of maximum signal for d‐bSSFP).

Metallic needles introduce a local inhomogeneity in the magnetic field. Following Ladd et al., we modeled the needle shaft as an infinite cylinder,^7^ placed along the y‐axis in a right‐handed coordinate system (Figure 5A). For a magnetic field $B_{0}=\left(0,B_{0}\;\cos\left(\theta \right),B_{0}\;\sin\left(\theta \right)\right)$ that encloses an angle *θ* with the y‐axis, the local change in magnetic field magnitude Δ*B*_susc_ is given by

(4) $$\Delta B_{\text{susc}}\left(x,z\right)=B_{0}R^{2}\frac{\left(\chi_{e}-\chi_{i}\right)}{2}\sin^{2}\left(\theta \right)\left(\frac{x^{2}-z^{2}}{{\left(x^{2}+z^{2}\right)}^{2}}\right),$$

where *χ*_i_ is the magnetic susceptibility of the needle material itself, whereas *χ*_e_ is the susceptibility of the surrounding material, and *R* is the radius of the biopsy needle. Calculating the derivative of Eq. (4), we can derive the local susceptibility‐induced magnetic field gradient moment as follows:

(5) $$m_{0,\text{susc}}=\nabla \left(\Delta B_{\text{susc}}\right)\mathrm{TE}=B_{0}R^{2}\frac{\chi_{e}-\chi_{i}}{2}\sin^{2}\left(\theta \right)\mathrm{TE}\left(\begin{array}{c} \frac{2x\left(-3z^{2}+x^{2}\right)}{{\left(z^{2}+x^{2}\right)}^{3}}\\ 0\\ \frac{-2z\left(z^{2}-3x^{2}\right)}{{\left(z^{2}+x^{2}\right)}^{3}} \end{array}\right).$$

We additionally introduce a WM gradient moment acting on the local magnetization at TE as follows:

(6) $$m_{0,\mathrm{WM}}=\left(\begin{array}{c} 0\\ m_{0,\mathrm{WM}}\cos\left(\theta \right)\\ m_{0,\mathrm{WM}}\sin\left(\theta \right) \end{array}\right).$$

As described in Section 2, maximum WM signal for d‐FLASH and d‐SSFP contrast will be reached for points where susceptibility‐induced and WM gradient moments have the same magnitude but opposite polarity. Hence, maximum signal magnitude will be reached when $m_{0,\text{susc},x}=0$, as any susceptibility‐induced gradient moment in x ‐direction cannot be compensated by a WM gradient as defined in Eq. (6), and the needle‐induced gradient moment magnitude is fully compensated by the WM gradient moment: $m_{0,\text{susc},z}=-m_{0,\mathrm{WM},z}$ (the y‐component of $m_{0,\mathrm{WM}}$ induces a global dephasing and will always be non‐zero for needle orientations with a component parallel to $B_{0}$). For d‐bSSFP contrast, maximum WM signal is equivalently reached for points where susceptibility‐induced and WM gradient have the same magnitude and polarity: $m_{0,\text{susc},z}=m_{0,\mathrm{WM},z}$. The criterion for maximum WM signal can therefore be expressed by the following system of equations:

(7) $$\begin{array}{ll} & B_{0}R^{2}\frac{\chi_{e}-\chi_{i}}{2}\sin^{2}\left(\theta \right)\mathrm{TE}\left(\frac{2x\left(-3z^{2}+x^{2}\right)}{{\left(z^{2}+x^{2}\right)}^{3}}\right)=0\\ & B_{0}R^{2}\frac{\chi_{e}-\chi_{i}}{2}\sin^{2}\left(\theta \right)\mathrm{TE}\left(\frac{-2z\left(z^{2}-3x^{2}\right)}{{\left(z^{2}+x^{2}\right)}^{3}}\right)\pm m_{0,\mathrm{WM}}\sin\left(\theta \right)=0. \end{array}$$

We solve Eq. (7) for a WM gradient induced phase of $\gamma m_{0,\mathrm{WM}}=2\pi /\Delta z$. While the condition $m_{0,\text{susc},x}=0$ is fulfilled for z=0x and $z=\pm \sqrt{3}x$, we find $|m_{0,\mathrm{susc}}|=m_{0,\mathrm{WM},z}$ to be true on a concentric circle in the zx‐plane with radius r as follows:

(8) $$r=\sqrt[{3}]{\frac{\sin^{2}\left(\theta \right)\mathrm{TE}\;\gamma B_{0}R^{2}\left(\chi_{e}-\chi_{i}\right)\Delta z}{2\pi }}$$

We therefore find the location of the WM maxima in the zx‐plane as follows:

(9) $$\begin{array}{ll} X_{1,2} & =\left(z_{1,2},x_{1,2}\right)=\left(\pm \sqrt[{3}]{\frac{\sin\left(\theta \right)\mathrm{TE}\;\gamma B_{0}R^{2}\left(\chi_{e}-\chi_{i}\right)\Delta z}{2\pi }},0\right)\\ X_{3,4} & =\left(z_{3,4},x_{3,4}\right)=\left(\pm \sqrt[{3}]{\frac{\sin\left(\theta \right)\mathrm{TE}\;\gamma B_{0}R^{2}\left(\chi_{e}-\chi_{i}\right)\Delta z}{16\pi }},\right.\\ & \;\left.\sqrt{3}\sqrt[{3}]{\frac{\sin\left(\theta \right)\mathrm{TE}\;\gamma B_{0}R^{2}\left(\chi_{e}-\chi_{i}\right)\Delta z}{16\pi }}\right)\\ X_{5,6} & =\left(z_{5,6},x_{5,6}\right)=\left(\pm \sqrt[{3}]{\frac{\sin\left(\theta \right)\mathrm{TE}\;\gamma B_{0}R^{2}\left(\chi_{e}-\chi_{i}\right)\Delta z}{16\pi }},\right.\\ & \;\left.-\sqrt{3}\sqrt[{3}]{\frac{\sin\left(\theta \right)\mathrm{TE}\;\gamma B_{0}R^{2}\left(\chi_{e}-\chi_{i}\right)\Delta z}{16\pi }}\right). \end{array}$$

Figure 5B shows the induced local gradient field for a cylindrical needle in the zx‐plane and the WM gradient field as an overlay. The x‐component of the susceptibility‐induced gradient moment vanishes along the dashed black lines. Along the solid black line with the radius r, the magnitude of the susceptibility‐induced gradient moment is equal to the magnitude of the z‐component of the WM gradient moment. Three intersection points ($X_{1},X_{4},X_{6}$) can be identified where the susceptibility‐induced gradient moment has the opposite polarity of the WM gradient moment (yellow triangles). At these points, highest WM signal can be achieved for d‐FLASH, d‐SSFP and d‐bSSFP. Three additional points ($X_{2}$, $X_{3}$, $X_{5}$) can be identified, where gradients have the same polarity (yellow crosses). At these points, additional WM signal maxima in the zx‐plane are expected for d‐bSSFP. In three dimensions, the WM needle artifact therefore consists of either three or six bright stripes flanking the needle shaft. For the maximum signal magnitude, a ratio of about 0.64 between d‐bSSFP and d‐SSFP contrast is expected (see Eq. [A1]). Due to the higher symmetry of the d‐bSSFP needle artifact compared with the d‐SSFP artifact, the d‐bSSFP artifact contains twice as many pixels with maximum signal; thus, we expect a cumulative signal magnitude ratio twice as large (≈1.27). For a comparison of d‐FLASH with d‐SSFP and d‐bSSFP, a general prediction of the maximum signal magnitude ratio or the cumulative signal magnitude ratio is not feasible, as two different steady states are reached (coherent for d‐SSFP and d‐bSSFP, incoherent for d‐FLASH). While the maximum signal magnitude for the incoherent steady‐state signal depends on T_1_ and TR (maximized for Ernst angle), maximum coherent steady‐state signal magnitude is T_1_‐dependent and T_2_‐dependent,^31^ making the signal intensity ratio between d‐FLASH and d‐SSFP, as well as between d‐FLASH and d‐bSSFP, tissue‐dependent.

### 3.3 Measurements

To verify the introduced model and compare the different investigated contrast techniques, a 20‐gauge (*R* = 0.04059 cm) MR‐compatible aspiration needle (Cook Medical, Bloomington, IN, USA), made from Inconel (Nickel‐Chromium alloy with magnetic susceptibility *χ*_i_ = 568 × 10^−6^),^32^ was placed in a flask phantom (at an angle *θ* = 90° to $B_{0}$ as defined in Figure 5A) filled with deionized water (magnetic susceptibility *χ*_e_ = −9.05 × 10^−6^).^33^ Images with d‐FLASH, d‐SSFP, and d‐bSSFP contrast were acquired using the implemented sequences (see Section 3.1). The phantom was imaged at *B*_0_ = 0.55 T (MAGNETOM Free.Max; Siemens Healthineers, Erlangen, Germany) using a 12‐channel head coil and the following sequence parameters: field of view = (128 mm)^3^; res = (2 mm)^3^; TR = 40 ms; TE = 20 ms, bandwidth = 900 Hz/px, flip angle = 70° (d‐SSFP, d‐bSSFP) / 10° (d‐FLASH, Ernst angle as determined from a parameter sweep with step size of 2°). An additional reference image of the needle phantom, using a spin‐echo sequence, was acquired with the following sequence parameters: field of view = (128 mm)^3^; res = (1 mm)^3^; TE = 152 ms; TR = 3000 ms; bandwidth = 501 Hz/px. Maximum artifact signal magnitude (averaged across 3 or 6 points of maximum artifact signal magnitude in the zx‐plane for a slab of 10 slices) was compared between the different contrast mechanisms and, for the ratio between d‐bSSFP and d‐SSFP, compared with the theoretical prediction (see Section 3.2). Additionally, the cumulated artifact signal magnitude was evaluated (summed signal of 3 or 6 points of maximum artifact signal magnitude in the zx‐plane, averaged across a slab of 10 slices) and, for d‐SSFP and d‐bSSFP, again compared with the theoretically expected ratio. To further evaluate the accuracy of the model, d‐bSSFP contrast was acquired with varying TE (TE_1_ = 3.4 ms, TE_2_ = 10 ms, TE_3_ = 20 ms, TE_4_ = 30 ms, TE_5_ = 40 ms, TE_6_ = 50 ms), and the radius of the needle artifact was determined from the images by averaging over the distance from the true needle position as extracted from the spin‐echo reference and the position of the pixels with maximum artifact magnitude (assumed measurement uncertainty of one pixel, corresponding to half of the resolution). The measured artifact radius was then compared with the artifact radius predicted from Eq. (8) to evaluate the model's accuracy.

## 4 RESULTS

**FIGURE 6:**
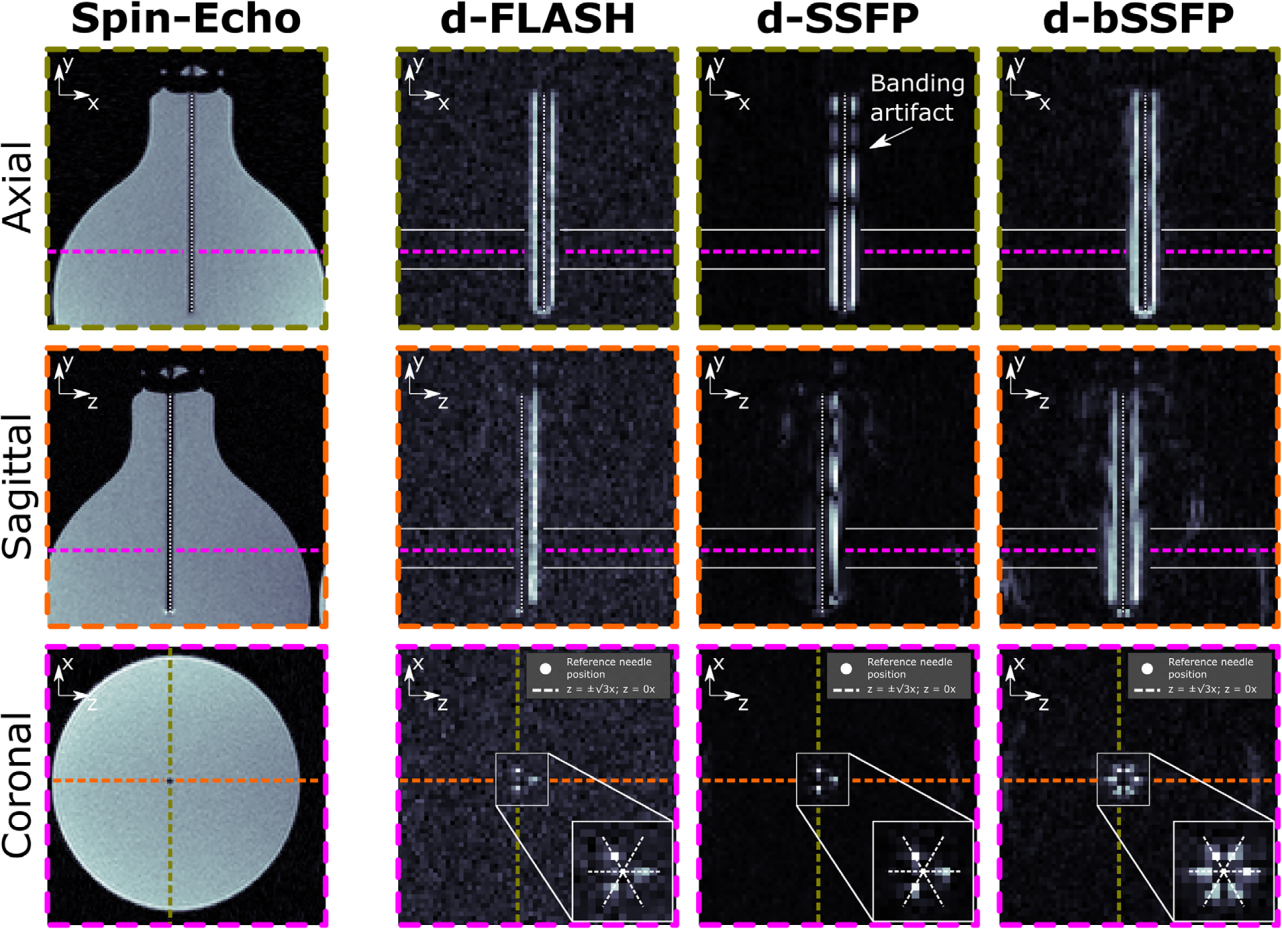
A 20‐gauge needle was placed in a phantom bottle filled with deionized water (orthogonal to $B_{0}$). A reference spin‐echo image (xy‐plane, zy‐plane, and zx‐plane as defined in Figure 5A; needle axis indicated by dotted line) as well as images acquired with d‐FLASH, d‐SSFP, and d‐bSSFP are shown (xy‐plane view is slightly offset from the needle axis for the dephased contrast compared with the reference to depict the artifact). An additional zoomed view on the artifact is displayed for the zx‐plane view. For a slab of 10 slices (between white solid lines drawn in the xy‐plane and zy‐plane images), we calculated the mean signal averaged across the three, respectively six, pixels with maximum artifact signal in each slice (see Table 2).

Figure 6 shows a reference spin‐echo image of the flask phantom, along with the d‐FLASH, d‐SSFP, and d‐bSSFP images. While for d‐FLASH and d‐SSFP only three signal maxima emerge in the zx‐plane (three stripes flanking the needle shaft, axisymmetric artifact with respect to z‐axis), six maxima (point‐symmetric artifact) are visible for d‐bSSFP. While the artifact appears spatially homogeneous for d‐FLASH and d‐bSSFP, multiple dark bands appear in d‐SSFP that break up the artifact (banding).

The maximum artifact magnitude for d‐bSSFP and d‐FLASH was found to be (0.56 ± 0.09) and (0.19 ± 0.04) relative to d‐SSFP (Figure 6). The theoretical signal ratio for d‐bSSFP and d‐SSFP (≈0.64) is therefore found to be within one standard deviation of the experimental value. The measured cumulated signal magnitude for d‐bSSFP and d‐FLASH was (1.13 ± 0.07) and (0.19 ± 0.01) relative to d‐SSFP. The theoretical cumulative signal ratio for d‐bSSFP and d‐SSFP (≈1.27) is therefore found to be within two standard deviations of the experimental value. Directly comparing d‐bSSFP and d‐FLASH, d‐bSSFP exhibits a (2.9 ± 0.5) and a (5.9 ± 0.4) times larger maximum artifact signal and cumulated signal magnitude than d‐FLASH.

**FIGURE 7:**
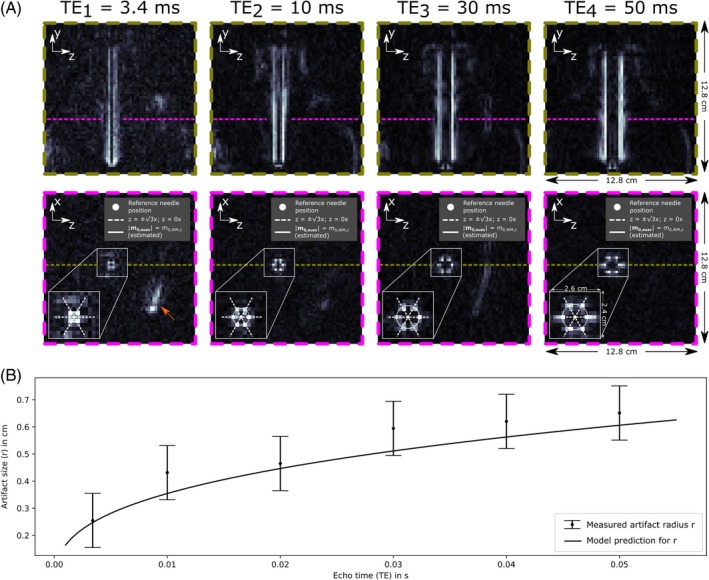
(A) The needle phantom (compare with Figure 6) was imaged with the d‐bSSFP sequence at six different echo times: TE_1_ = 3.4 ms, TE_2_ = 10 ms, TE_3_ = 20 ms, TE_4_ = 30 ms, TE_5_ = 40 ms, and TE_6_ = 50 ms. Slices corresponding to the yz‐plane and zx‐plane of the needle coordinate system, as defined in Figure 5A, are displayed for TE_1,2,4,6_. An additional zoomed view on the artifact is displayed for the zx‐plane view. The estimated location where $|m_{0,\mathrm{susc}}|=m_{0,\mathrm{WM},z}$ (compare with Figure 5B) is indicated with a white circle with the radius r, calculated as the average distance between the reference needle position and pixels of maximum signal. Some stripe‐like artifacts (orange arrow) can be seen in the water phantom (see discussion in Section 5). (B) The measured radius is plotted against TE (with half‐pixel measurement uncertainty). Additionally, the modeled radius from Eq. (8) is plotted using the literature values for the magnetic susceptibility of Inconel and water.

Figure 7 compares the needle artifact for different TEs. d‐bSSFP images for four example TEs are shown in Figure 7A. Some stripe‐like artifacts can be seen in the water phantom that appear unrelated to the needle (see discussion in Section 5). Needle WM artifact size increased with increasing TE. The true location of the needle shaft was extracted from the reference spin‐echo image and added to the d‐bSSFP images (white dot). Dashed and solid lines represent the presumed location of vanishing $m_{0,\text{susc},x}$ and $|m_{0,\mathrm{susc}}|=m_{0,\mathrm{WM},z}$, respectively, according to the introduced model and corresponding to Figure 5B. Pixels with maximum intensity can be seen to lie on the dashed lines with a maximum deviation of one pixel. The measured artifact radius was plotted against TE in Figure 7B. The artifact radius as predicted by the model is also plotted. The measured artifact radius was in accordance with the model (within measurement uncertainty) for all investigated TEs.

Results, comparing the three investigated contrast types (exerted artifact symmetry, sensitivity to banding artifacts, theoretically expected and measured signal magnitude), are summarized in Table 2.

**TABLE 2:** Comparison of the investigated contrast mechanisms (d‐FLASH, d‐SSFP, and d‐bSSFP) comprising the exhibited symmetry, the sensitivity to banding artifacts, and the maximum as well as the cumulated artifact signal magnitude (normalized to d‐SSFP magnitude for theoretical model prediction and measurements, given with standard deviation).

	d‐FLASH	d‐SSFP	d‐bSSFP
Symmetry	Axial	Axial	Point
Sensitive to banding artifacts	No	Yes	No
Maximum artifact magnitude (normalized to d‐SSFP)	Model: n.a.	Model: 1	Model: 0.64
	Exp.: 0.19 ± 0.04	Exp.: 1.0 ± 0.1	Exp.: 0.56 ± 0.09
Cumulated artifact magnitude (normalized to d‐SSFP)	Model: n.a.	Model: 1	Model: 1.27
	Exp.: 0.19 ± 0.01	Exp.: 1.00 ± 0.04	Exp.: 1.13 ± 0.07

## 5 DISCUSSION

In this work, d‐bSSFP was investigated as a positive, susceptibility‐based contrast method and compared with the related techniques d‐FLASH and d‐SSFP. A signal model was established and used to describe the artifact of a cylindrical metallic needle. Artifact shape, size, and signal magnitude as predicted by the model were compared with phantom measurements.

The d‐bSSFP signal model predicts, in contrast to d‐FLASH and d‐SSFP, a point‐symmetric artifact. Matching the theoretical result, the higher artifact symmetry was confirmed in phantom measurements and can be explained by the unique combination of a discrete and a continuous phase evolution, as demonstrated in Figure 3. In this paper, the artifact shape was evaluated for the example geometry of a metallic cylinder, but a point‐symmetric artifact is equally expected to emerge for spherical devices, such as fiducial markers, as they also induce a symmetric field distortion.^6^ A point‐symmetric artifact might provide some benefits for applications of a positive contrast technique, e.g., for localization tasks. The artifact appears more homogeneously around the device due to the additional symmetry (six instead of three signal maxima as shown in Figure 5), which might make it easier to detect the artifact center when viewing the images. The artifact center could, for example, be identified by simply connecting maximum signal points that are diagonally opposite of each other and taking the intersection. Also, the additional signal maxima in the d‐bSSFP artifact increase redundancy, which could, in particular, pose an advantage if the artifact is investigated in heterogeneous tissue instead of a homogeneous water phantom. As the focus of this paper remains the analysis of the d‐bSSFP contrast mechanism itself, further analysis of potential benefits in applications, such as localization tasks, shall be addressed in future work.

As described in Section 3.1, the three investigated research pulse sequences (d‐FLASH, d‐SSFP, and d‐bSSFP) were implemented to follow a radial acquisition scheme, as it is inherently immune to spatial signal shifts that can occur in the proximity to magnetic disturbers. For a potential Cartesian implementation, any possible distortions of the artifacts could be counteracted by deliberately choosing a high receiver bandwidth. The WM gradient moment in the investigated pulse sequences was implemented such that it acted in the $B_{0}$ direction. In general, a different direction for the WM gradient moment could also be chosen, and the position of the artifact signal maxima could be similarly determined from an adapted Eq. (7). Although orientation of the artifact can change with the direction of the WM gradient moment, no fundamental change in the artifact's general appearance and symmetry is expected, as the investigated signal model is independent of the WM orientation.

While in Figure 6 the artifact appears homogeneous for d‐FLASH and d‐bSSFP, dark banding artifacts interrupt the artifact for d‐SSFP. This sensitivity of the d‐SSFP contrast to banding can be explained from the mechanism of contrast formation. For d‐SSFP, the unbalanced susceptibility‐induced gradient is counterbalanced by an equally unbalanced external WM gradient as described in Section 2. At points of maximum gradient compensation, no net gradient remains, and the sequence therefore mimics the classic balanced SSFP sequence type. The signal can be said to locally “morph” itself into a bSSFP echo.^15^, ^34^ This automatically makes the artifact prone to banding artifacts that appear in bSSFP. In d‐bSSFP, banding artifacts are not an issue, as the local magnetization at points of maximum signal compensation experiences an unbalanced susceptibility gradient purposely introducing a phase flip in all voxels. A much smaller background gradient due to imperfect shimming will only lead to a negligible change in the unbalanced gradient. The sensitivity to banding puts d‐SSFP at a disadvantage compared with d‐FLASH and d‐bSSFP, as artifact homogeneity is crucial for the visualization and delineation of a device.

For d‐SSFP and d‐bSSFP, the ratio of the maximum artifact signal and the cumulated artifact signal was theoretically determined using the introduced models. Due to the different types of established steady‐state magnetization ($S_{\mathrm{SSI}}$ and $S_{\mathrm{SSC}}$), the maximum signal magnitude ratio between d‐FLASH and d‐SSFP, as well as d‐FLASH and d‐bSSFP, is tissue dependent, and a general prediction is not feasible (see Section 3.2). For all three investigated contrast methods, maximum and cumulated signal magnitude were compared experimentally. Generally, a higher signal level can be reached with SSFP imaging techniques than with RF‐spoiled GRE (FLASH) imaging techniques.^31^, ^35^ This is reflected accordingly by the measured maximum artifact signal magnitude being lowest for d‐FLASH (Table 2). For d‐bSSFP, a slightly lower maximum (within one standard deviation) and cumulative signal ratio (within two standard deviations) were measured experimentally than were predicted by the model. This observation can potentially be attributed to the model's assumptions of a spatially constant gradient and a constant coherent steady‐state signal $S_{\mathrm{SSC}}$ independent of the locally induced phase advance during TR, which are not perfectly matched by reality. Equation (2) furthermore assumes a uniform voxel sensitivity function. Due to the nature of the discrete Fourier transform used in MRI, imaged magnitude is however not sampled equally from the original distribution of transverse magnetization throughout the voxel.^36^ This might cause an additional deviation of the measurement from the model in terms of a reduced measured signal magnitude, as the measured signal might not fully represent a phase coherence that is established by the WM gradient across the complete voxel. However, the cumulated artifact signal being highest for d‐bSSFP, as theoretically predicted, was experimentally confirmed and might, similar to the higher artifact symmetry, prove useful in potential applications of the positive contrast, as it could improve visibility of devices or SPIO‐labeled cells.

For further validation of the signal model, d‐bSSFP needle artifact size was investigated for six different TEs. For the investigated TEs, the measured artifact radius was well in accordance with the curve in Figure 7B representing the relationship in Eq. (8), as all measured values matched the predicted radius within the margin of error. Stripe‐like artifacts in the water phantom are visible in some of the d‐bSSFP images in Figure 7A. The origin of these artifacts, which appear to be unrelated to the needle due to the spatial distance, is not clear, but they could be eddy current–related and emerge due to the applied radial readout trajectory.^37^ In this case, a reduction in artifact magnitude would be expected for a Cartesian readout trajectory.

To evaluate the different contrast methods, signal models for d‐FLASH, d‐SSFP, and d‐bSSFP were investigated. While the signal magnitude for d‐FLASH is described by the well‐known sinc relation,^10^ the investigated signal models for d‐SSFP and d‐bSSFP have, to the best of our knowledge, not yet been introduced in the literature. As described in Section 2, the models predict the signal for a pixel that experiences a certain dephasing due to spoiled or balanced and unspoiled/unbalanced gradients acting on the local magnetization either solely or jointly. Due to the discrete nature of the established phase for unspoiled/unbalanced gradients, the d‐SSFP and d‐bSSFP signal also depend on the local discrete phase offset, and we calculated an average voxel signal magnitude. For a spatially‐constant unspoiled/unbalanced gradient, a modulation of signal magnitude across multiple voxels, depending on the local phase offset, would be expected (appearance of bright bands for d‐bSSFP that appear closer to each other for more effective gradient compensation). Effects of this discrete behavior could, however, not be seen in the conducted experiments. As, for the investigated needle geometry, the magnitude of the susceptibility‐induced gradient decreases quickly (with $\frac{1}{r^{3}}$ as derived from Eq. [4]) with increasing distance from the device, no bands are formed.

In this work, we studied d‐bSSFP contrast and compared it with the related contrast techniques d‐FLASH and d‐SSFP. A signal model for d‐bSSFP was established and used for artifact prediction of a cylindrical aspiration needle. Compared with the related techniques d‐FLASH and d‐SSFP, we found the d‐bSSFP needle artifact to present with higher artifact symmetry and higher cumulated artifact signal. Compared with the d‐SSFP artifact, the d‐bSSFP artifact showed robustness against banding artifacts. The properties of the d‐bSSFP contrast encourage further studies to investigate the application of the contrast technique in interventional MRI and potentially in imaging of SPIO‐labeled cells or fiducial marker localization, as they could render d‐bSSFP beneficial compared with d‐FLASH and d‐SSFP for delineation and localization tasks.

## CONFLICT OF INTEREST

Jonas Frederik Faust, Peter Speier, Axel Joachim Krafft, Sunil Patil, Ravi Teja Seethamraju, and Florian Maier are employees of Siemens Healthineers.

## APPENDIX A

### A.1

For the signal equations set up in Eq. (3), we can find closed solutions. For the d‐FLASH signal equation, the solution is the well‐known sinc function.^10^ For d‐SSFP and d‐bSSFP, we can determine the solution to the signal equations using geometrical arguments (here given up to first signal maximum at $\gamma m_{0,\text{susc}}=\pm 2\pi /\Delta z$ for ${\tilde{S}}_{d‐\text{bSSFP}}$):

(A1)
$$\begin{array}{ll} {\tilde{S}}_{d‐\text{FLASH}} & =S_{\mathrm{SSI}}\left|\int \text{rect}\left(\frac{z}{\Delta z}\right)e^{-i\left(\gamma m_{0,\text{susc}}-\frac{2\pi }{\Delta z}\right)z}dz\right|\\ & =S_{\mathrm{SSI}}\left|\mathrm{sinc}\left(\gamma m_{0,\text{susc}}\Delta z/2-\pi \right)\right|\\ {\tilde{S}}_{d‐\text{SSFP}} & =S_{\mathrm{SSC}}\frac{\int_{0}^{2\pi }\left|\int \text{rect}\left(\frac{z}{\Delta z}\right)e^{-i\;\mathrm{SW}\left(\left(\gamma m_{0,\text{susc}}-\frac{2\pi }{\Delta z}\right)z+\tau \right)}dz\right|d\tau }{2\pi }\\ & =S_{\mathrm{SSC}}\left\{\begin{array}{ll} 1 & \text{for}\;\gamma m_{0,\text{susc}}\Delta z=-2\pi \\ \frac{\mathrm{tri}\left(\mathrm{mod}\left(\gamma m_{0,\text{susc}}\Delta z,2\pi \right)/\pi -1\right)}{\left|\gamma m_{0,\text{susc}}\Delta z+2\pi \right|/\pi }\left(1-\frac{\mathrm{tri}\left(\mathrm{mod}\left(\gamma m_{0,\text{susc}}\Delta z,2\pi \right)/\pi -1\right)}{2}\right) & \text{for}\;\gamma m_{0,\text{susc}}\Delta z\neq -2\pi \end{array}\right.\\ {\tilde{S}}_{d‐\text{bSSFP}} & =S_{\mathrm{SSC}}\frac{\int_{0}^{2\pi }\left|\int \text{rect}\left(\frac{z}{\Delta z}\right)e^{-i\;\left(\mathrm{SW}\left(\gamma m_{0,\text{susc}}z+\tau \right)-\frac{2\pi }{\Delta z}z\right)}dz\right|d\tau }{2\pi }\\ & =S_{\mathrm{SSC}}\left\{\begin{array}{ll} \frac{4}{\pi^{3}}\left|\gamma m_{0,\text{susc}}\Delta z\right| & \text{for}\;\left|\gamma m_{0,\text{susc}}\Delta z\right|<\pi \\ \begin{array}{l} \frac{2\left|\gamma m_{0,\text{susc}}\Delta z\right|}{\pi^{3}}\left(\cos\left(\frac{\pi }{\gamma m_{0,\text{susc}}\Delta z}\mathrm{mod}\left(\left|\gamma m_{0,\text{susc}}\Delta z\right|,\pi \right)\right)-\cos\left(\frac{\pi^{2}}{\gamma m_{0,\text{susc}}\Delta z}\right)\right)\\ +\frac{2}{\pi^{2}}\sin\left(\frac{\pi^{2}}{\left|\gamma m_{0,\text{susc}}\Delta z\right|}\right)\mathrm{mod}\left(\left|\gamma m_{0,\text{susc}}\Delta z\right|,\pi \right) \end{array} & \text{for}\;\pi \leq \left|\gamma m_{0,\text{susc}}\Delta z\right|<2\pi \\ \frac{2}{\pi } & \text{for}\;\left|\gamma m_{0,\text{susc}}\Delta z\right|=2\pi . \end{array}\right. \end{array}$$

Here, rect is the rectangular function (rect(x)=1 for |x|<0.5; rect(x)=0.5 for x=±0.5; rect(x)=0 elsewhere); tri is the triangular function (tri(x)=max(1−|x|,0)); and mod(a,b) describes the modulo operation. We therefore find ${\tilde{S}}_{d‐\text{bSSFP}}\left(\gamma m_{0,\text{susc}}=\pm 2\pi /\Delta z\right)\approx 0.64S_{\mathrm{SSC}}$ for the case of maximum signal magnitude.
